# AI-based quantification of inflammatory extent for relapse prediction in ulcerative colitis: a prospective cohort study

**DOI:** 10.1093/ecco-jcc/jjag115

**Published:** 2026-07-30

**Authors:** Yasuharu Maeda, Shin-Ei Kudo, Noriyuki Ogata, Kento Takenaka, Kaoru Takabayashi, Takanori Kuroki, Yurie Kawabata, Taishi Okumura, Tatsuya Sakurai, Yuta Kouyama, Katsuro Ichimasa, Takemasa Hayashi, Toshiyuki Baba, Haruhiko Ogata, Kazuo Ohtsuka, Yuichi Mori, Marietta Iacucci, Masashi Misawa

**Affiliations:** Digestive Disease Center, Showa Medical University Northern Yokohama Hospital, Yokohama, Kanagawa, 224-8503, Japan; Digestive Disease Center, Showa Medical University Northern Yokohama Hospital, Yokohama, Kanagawa, 224-8503, Japan; Digestive Disease Center, Showa Medical University Northern Yokohama Hospital, Yokohama, Kanagawa, 224-8503, Japan; Department of Gastroenterology and Hepatology, Institute of Science Tokyo, Tokyo, 113-8519, Japan; Center for Diagnostic and Therapeutic Endoscopy, Keio University School of Medicine, Tokyo, 160-8582, Japan; Digestive Disease Center, Showa Medical University Northern Yokohama Hospital, Yokohama, Kanagawa, 224-8503, Japan; Digestive Disease Center, Showa Medical University Northern Yokohama Hospital, Yokohama, Kanagawa, 224-8503, Japan; Digestive Disease Center, Showa Medical University Northern Yokohama Hospital, Yokohama, Kanagawa, 224-8503, Japan; Digestive Disease Center, Showa Medical University Northern Yokohama Hospital, Yokohama, Kanagawa, 224-8503, Japan; Digestive Disease Center, Showa Medical University Northern Yokohama Hospital, Yokohama, Kanagawa, 224-8503, Japan; Digestive Disease Center, Showa Medical University Northern Yokohama Hospital, Yokohama, Kanagawa, 224-8503, Japan; Digestive Disease Center, Showa Medical University Northern Yokohama Hospital, Yokohama, Kanagawa, 224-8503, Japan; Digestive Disease Center, Showa Medical University Northern Yokohama Hospital, Yokohama, Kanagawa, 224-8503, Japan; Center for Preventive Medicine, Keio University, Tokyo, 106-0041, Japan; Fujita Medical Innovation Center Tokyo, Tokyo, 144-0041, Japan; Endoscopy Unit, Institute of Science Tokyo, Tokyo, 113-8519, Japan; Clinical Effectiveness Research Group, Institute of Health and Society, University of Oslo, Oslo, 0317 Norway; APC Microbiome Ireland, College of Medicine and Health, University College Cork, Cork, T12 YT20, Ireland; Digestive Disease Center, Showa Medical University Northern Yokohama Hospital, Yokohama, Kanagawa, 224-8503, Japan

**Keywords:** deep learning, endoscopic remission, relapse prediction

## Abstract

**Background and Aims:**

Endoscopic remission is a key therapeutic goal in ulcerative colitis (UC); however, conventional indices focus on peak severity without accounting for the spatial extent of inflammation. We developed a deep learning-based score, Quantitative Ulcerative Colitis Assessment using Deep Learning (QUAD), and evaluated whether incorporating inflammatory extent improves relapse prediction in patients with UC in clinical remission. To our knowledge, no studies have examined whether artificial intelligence (AI)-derived assessment of inflammatory extent predicts clinical relapse.

**Methods:**

This prospective cohort study evaluated relapse prediction over 24 months in patients with UC in clinical remission. The QUAD model assigns a score of 0-3 to each image quadrant, yielding a total score of 0-12. The model was trained on 84 743 images from 998 patients with UC across three centers. Still image-based validation and automated full-length video analysis were conducted to assess the impact of inflammatory extent on relapse prediction.

**Results:**

Clinical relapse occurred in 19.4% of patients with QUAD ≥ 4 compared with 5.2% of those with QUAD < 4 (*P* = .01), with an area under the curve (AUC) of 0.67 (95% confidence interval [CI]: 0.57-0.76). Notably, automated video-based analysis showed that distal inflammatory burden yielded the highest predictive performance, with analysis of the distal 10% segment achieving an AUC of 0.73 (95% CI: 0.62-0.85), compared with whole-colon assessment (AUC, 0.62; 95% CI: 0.48-0.76) and still image-based evaluation.

**Conclusion:**

AI-augmented assessment integrating inflammatory severity and extent may provide complementary prognostic information beyond severity-based evaluation.

## 1. Introduction

Ulcerative colitis (UC) is a chronic inflammatory bowel disease marked by alternating periods of remission and relapse.^1^^,^^2^ Achieving and maintaining endoscopic remission through appropriate pharmacological treatment is essential because it supports long-term clinical remission, reduces the likelihood of surgery for management of recurrent inflammation, and lowers the risk of colitis-associated colorectal cancer.^3^^,^^4^

With treat-to-target strategies now standard in UC management, endoscopic remission is widely recognized as a key long-term goal.^5^^,^^6^ Conventional endoscopic indices, such as the Mayo Endoscopic Subscore (MES),^7^ focus primarily on the most severely affected mucosal areas.^7^ However, recent evidence indicates that both the severity and extent of mucosal inflammation have important prognostic value.^8^^,^^9^

Advances in artificial intelligence (AI) have enabled automated endoscopic assessment of UC, including approaches that incorporate not only image-level severity^10^^,^^11^ but also classification of inflammatory extent.^12^^,^^13^ Despite these methodological advances, the clinical utility of incorporating inflammatory extent into relapse prediction remains unclear.^14^ To address this limitation, we developed the Quantitative Ulcerative Colitis Assessment using Deep Learning (QUAD), a quadrant-based AI score designed to capture both the severity and extent of inflammation.

The aim of this prospective cohort study was to evaluate whether QUAD predicts clinical relapse in patients with UC in clinical remission and to explore whether still image-based and video-based assessments provide prognostic information.

## 2. Methods

This study comprised two main phases: multicenter AI model development (Phase I) and prospective validation (Phase II). Phase II included two analytic components: still image-based validation and automated full-length video analysis.

**Figure 1.**
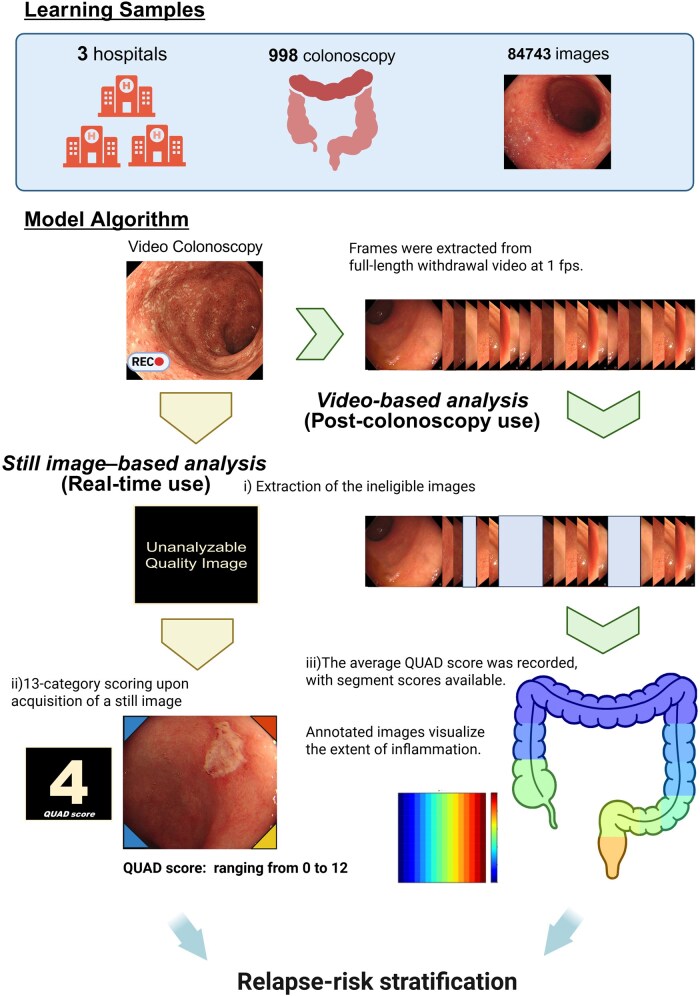
Overview of the Quantitative Ulcerative Colitis Assessment using Deep Learning (QUAD) framework and its clinical application. The QUAD system can be used in two complementary ways. During colonoscopy, still image-based analysis provides a real-time QUAD score reflecting inflammatory severity. After colonoscopy, automated video analysis quantifies inflammatory extent throughout the examined bowel. Both outputs may be used for relapse-risk stratification in patients with ulcerative colitis. Created with https://BioRender.com

We developed and prospectively validated a quantitative, AI-driven colonoscopic scoring system, ranging from 0 to 12, called QUAD (Figure 1). The QUAD algorithm was trained using endoscopic data from three centers: Keio University Hospital, the Institute of Science Tokyo Hospital, and Showa Medical University Northern Yokohama Hospital. The validation study comprised three sequential components. First, we assessed the feasibility of the QUAD algorithm by examining its correlation with endoscopic disease activity in patients with UC. Second, we performed a single-group, open-label, prospective study to evaluate whether a QUAD score derived from still images could predict clinical relapse in patients with UC. Third, we applied automated video analysis to full-length withdrawal videos from the same cohort to quantify inflammatory extent. We then evaluated whether the AI-quantified inflammatory extent was associated with relapse. The primary endpoint was clinical relapse within 24 months. Secondary analyses included time-to-relapse assessment and exploratory video-based evaluation of inflammatory extent.

### 2.1. System description

The development of our QUAD system utilized the capabilities of a convolutional neural network, using the 16-layer Visual Geometry Group (VGG16) architecture. The algorithm was trained on 84 743 colonoscopic images from 998 patients with UC, collected between October 2016 and June 2022 across Keio University Hospital, the Institute of Science Tokyo Hospital, and Showa Medical University Northern Yokohama Hospital. Each colonoscopic image was divided vertically and horizontally to produce four quadrant (¼-frame) images. Two board-certified gastrointestinal endoscopists—each with experience in more than 20 000 colonoscopies—independently scored every quadrant according to a modified MES. Quadrants showing normal mucosa or scar tissue were assigned a score of 0, and those with erythema, yellow-white spots, or decreased vascular pattern received a score of 1. When erosion or mucosal granularity occupied ≥ 50% of the quadrant, a score of 2 was given; when an ulcer or spontaneous bleeding occupied ≥ 50% of the quadrant, a score of 3 was assigned. The set of quadrants labeled in this manner formed the training dataset for development of the QUAD-score algorithm. A QUAD score, ranging from 0 to 12, was calculated by summing the scores from the four quadrants in each image. Each quadrant was classified independently by the AI model, and the final QUAD score was calculated as the sum of the four quadrant-level predictions. A QUAD score of ≥ 4 was determined as the threshold for relapse prediction based on receiver operating characteristic curve analysis of data from a previous study.^11^ Low-quality frame exclusion was performed using a previously validated quality-control algorithm^11^ before QUAD scoring. The development dataset consisted of colonoscopic images collected between October 2016 and June 2022 from three institutions and was used exclusively for model training. In Phase I, an additional prospective dataset collected in July 2022 was used to assess the correlation between QUAD scores and MES but was not used for model training or threshold optimization. Prospective validation was subsequently conducted in an independent cohort enrolled between August 2022 and February 2023. No patient overlap was permitted among the development, Phase I assessment, and prospective validation cohorts.

This model offers two complementary workflows:

*Real-time mode*. During examination, every captured still image is automatically assigned a 13-class QUAD score, with the highest score serving as the representative value for relapse-risk stratification. The 13-class output corresponds to possible summed quadrant scores ranging from 0 to 12, representing all combinations of quadrant-level inflammation severity.

*Post-colonoscopy mode.* After the procedure, the full withdrawal video is uploaded to the system. To characterize the spatial distribution of inflammation, the software samples the video at one frame per second, automatically discarding poor-quality frames. Frame sampling at one frame per second was selected a priori to balance computational feasibility and representative coverage of mucosal evaluation. Each remaining frame is assigned a QUAD score, and the patient’s mean QUAD score is calculated and recorded.

Key features include:

- Automatic exclusion of low-quality images ineligible for analysis because of halation, inadequate air insufflation, close proximity to the mucosa, or the use of chromoendoscopy or virtual chromoendoscopy.
- Fully automated 13-class scoring based on frame-by-frame analysis. The model visualizes AI-detected inflammatory activity by displaying color-coded indicators (red, yellow, blue) in the four corners of the screen. This intuitive visual aid helps endoscopists locate specific regions of active inflammation identified by the AI.
- Processing of full-length withdrawal videos to quantify inflammatory extent. Frames are extracted at one frame per second, screened for quality, and scored according to QUAD. The average QUAD score is then calculated and recorded. In addition to generating scores for the entire colon, the system can output scores for user-specified segments. Simultaneously, visual representations of inflammation severity are displayed as annotated images on the output screen (Figure 1).

### 2.2. Colonoscopy procedure for validation study

Commercially available colonoscopes (CF-HQ290ZI, PCF-H290ZI, PCF-PQ260I/L, CF-XZ1200I, and CF-EZ1500DI; Olympus Medical Systems, Tokyo, Japan) were used in this study. For bowel preparation, patients ingested 2–4 L of polyethylene glycol solution on the morning of the examination. Clearance of intestinal fluid was verified before initiating the colonoscopy, which was performed under conscious sedation with intravenous diazepam (5–10 mg) or midazolam (2–10 mg).

#### 2.2.1. First phase

In July 2022, we prospectively collected endoscopic still images. The study included all patients with UC at our institution who underwent colonoscopy for clinical purposes and consented to participate. During withdrawal from the cecum, the endoscopists recorded still images according to clinical requirements, without observing the QUAD algorithm outputs. An expert endoscopist (N.O.) reviewed all recorded still images and assigned an MES. We then examined the correlations between the QUAD score and the MES.

#### 2.2.2. Second phase

##### 2.2.2.1. Still image-based analysis

A single-group, open-label, prospective study was conducted to evaluate whether a still image-based QUAD score could predict clinical relapse in patients with UC.

**Figure 2.**
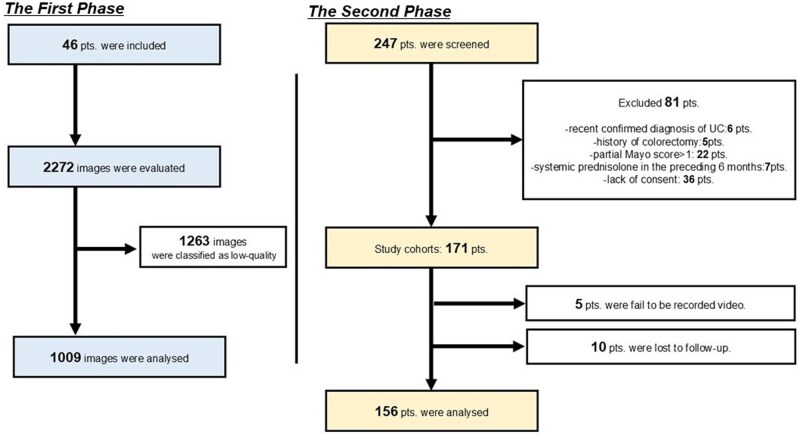
Study flow diagram across the three study components. In the first component, 46 patients provided 2272 images, of which 1009 high-quality images were analyzed. In the second and third components, 247 patients were screened; after exclusions and loss to follow-up, 156 patients were included in the final analysis.

*Study population*. Eligible patients with UC undergoing colonoscopy at Showa Medical University Northern Yokohama Hospital between August 2022 and February 2023 were prospectively enrolled. A flowchart of patient inclusion is shown in Figure 2.

*Inclusion criteria*. Patients were eligible if they had a confirmed diagnosis of UC and were in clinical remission at the time of colonoscopy.

*Exclusion criteria*. Patients were excluded if they had active UC (partial Mayo score of > 2), a history of colon surgery, or systemic prednisone use within 6 months before colonoscopy.

*Follow-up protocol*. Patients were followed in the outpatient clinic every 8–12 weeks for up to 24 months or until clinical relapse.

*Outcome definition*. The primary outcome measure was the rate of clinical relapse within 24 months after colonoscopy. Clinical relapse was defined as a partial Mayo score of > 2.

During withdrawal of the colonoscope from the cecum, the endoscopist captured at least one still image of the most severely inflamed area in each of the five colorectal segments (cecum–ascending colon, transverse colon, descending colon, sigmoid colon, and rectum) to generate diagnostic AI output. After each colonoscopy, the QUAD score for each image, as output by the AI, was recorded in a comma-separated file. One endoscopist (M.M.), blinded to the patient’s clinical course and endoscopic information, aggregated the AI outputs from the file and registered the AI-based QUAD assignments. Both patients and their outpatient clinicians were blinded to the AI output. Expert endoscopists (Y.M. and N.O.) entered each patient’s characteristics and clinical course data into the database. Patients were classified into two groups based on the QUAD score (<4 vs. ≥4) of the worst segment. The cutoff value of QUAD ≥ 4 was determined a priori using an independent dataset from our previous study^11^ before initiation of the present analysis and was not modified based on the current validation cohort. For segment scoring, when multiple images were available, the AI prediction was determined using the most frequently occurring QUAD score (eg, three outputs of QUAD score 0, two of score 1, and five of score 2 were classified as QUAD score 2). In cases of ties, the higher score was selected. The most frequent score was used to minimize the influence of outlier frames and to better represent the dominant inflammatory pattern within each segment.

##### 2.2.2.2. Video-based analysis

Because still image-based analysis relied on endoscopist-selected representative images, we also performed a separate automated video-based analysis to reduce frame-selection bias. Withdrawal videos were analyzed from cecal withdrawal to completion of the examination. Frames were extracted at 1 frame per second, and images classified as low quality by the predefined quality-control algorithm were excluded. For segmental analyses, the distal 50%, 40%, 30%, 20%, 10%, and 5% portions were defined according to withdrawal time and used as surrogate markers of progressively distal colonic segments. A schematic overview of the video segmentation workflow, including frame extraction, low-quality frame exclusion, segment definition, and mean QUAD score calculation, is provided in Figure S1.

We compared the area under the curve (AUC) of the score derived from the full video (100%-QUAD score) with scores based on only the distal 50%, 40%, 30%, 20%, 10%, and 5% of the video (50%-QUAD score through 5%-QUAD score, respectively) in predicting clinical relapse within 24 months, using the cohort from the second phase.

### 2.3. Statistical analysis

Statistical analysis of the primary endpoint was performed using a per-protocol set that excluded patients with missing data. All statistical analyses were performed using R statistical software (R Foundation for Statistical Computing, Vienna, Austria). Quantitative data were expressed as median (interquartile range) for continuous variables, and statistical significance was assessed using Fisher’s exact test, Student’s t-test, or the Mann–Whitney U test, as appropriate. A two-sided *P*-value of < .05 was considered statistically significant. The required sample size was estimated with reference to our previous study using AI-assisted colonoscopy. We assumed 24-month clinical relapse rates of 5% for patients with a QUAD score of < 4 and 30% for those with a QUAD score of ≥ 4, with a two-sided significance level of 0.05 and a power of 80%. The enrollment ratio of 1:2 for a QUAD score of < 4 versus ≥ 4 was based on an unpublished preliminary validation study using consecutive cases from our hospital. This calculation indicated that 45 and 90 patients would be required in the < 4 and ≥ 4 groups, respectively, at a 1:2 ratio. Allowing for patient dropouts, we determined that 160 participants would be needed.

### 2.4. Research enrollment and ethics

All patients provided informed consent for both the procedures and participation in the study. The Ethics Committee of Showa Medical University Northern Yokohama Hospital approved the study protocol (No. 21H018). The study was registered in the clinical trial registry of the University Hospital Medical Information Network (UMIN000049559) and conducted in accordance with the guidelines of the Declaration of Helsinki. All authors had full access to the study data and reviewed and approved the final manuscript.

## 3. Results

### 3.1. Model performance and correlation with endoscopic severity

**Figure 3.**
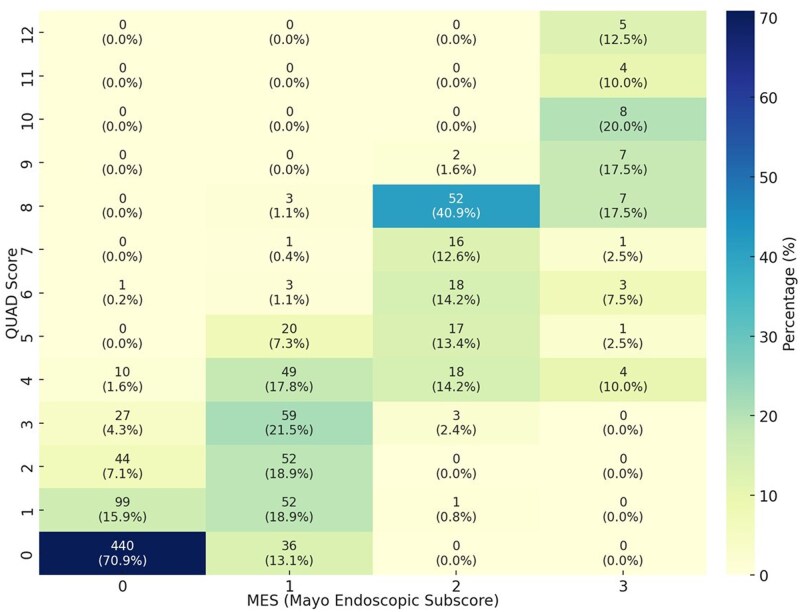
Heatmap of QUAD score distribution across MES categories. Each cell displays the number of cases and the corresponding percentage within each MES column. The vertical axis represents the QUAD score (0-12), with increasing severity from bottom to top. The color gradient reflects the relative proportion of each QUAD score within the respective MES group. MES, Mayo Endoscopic Subscore; QUAD, Quantitative Ulcerative Colitis Assessment using Deep Learning.

In the first phase, 2272 images from 46 patients were prospectively collected. Image quality assessment and exclusion of non-analyzable frames were performed automatically by the predefined AI pipeline. Of these, 1009 images met the predefined quality criteria and were included in the analysis. QUAD scores showed a strong correlation with MES (Spearman *r* = 0.809, *P* < .01) (Figure 3).

### 3.2. Prospective cohort characteristics

Among 252 screened patients, 156 (median age, 51 years; 49% male) were included in the prospective validation cohort (Figure 2). Baseline characteristics are shown in Table 1. During follow-up, clinical relapse occurred in 22 patients.

**Table 1.** Patient background characteristics.

	Overall (*n* = 156)	Non-relapse (*n* = 134)	Relapse (*n* = 22)	*P*-values
**Male, *n* (%)**	76 (48.7)	67 (50.0)	9 (40.9)	.50
**Age, years, median (IQR)**	51 (44-63)	52 (44-64)	50 (41-55)	.67
**Duration of disease, years, median (IQR)**	14 (7-19)	14 (8-20)	10 (7-19)	.12
**Extent of disease, *n* (%)**				.07
** Extensive colitis**	95 (60.9)	78 (58.2)	17 (77.3)	
** Left-sided colitis**	39 (25.0)	34 (25.4)	5 (22.7)	
** Proctitis**	22 (14.1)	22 (16.4)	0	
**Mayo Endoscopic Subscore, *n* (%)**				<.01
** 0**	72 (46.2)	69 (51.4)	3 (13.6)	
** 1**	65 (41.7)	53 (39.6)	12 (54.6)	
** 2-3**	19 (12.1)	12 (9.0)	7 (31.8)	
**Geboes histological index**				.03
** <3.1**	116 (74.4)	104 (77.6)	12 (54.6)	
** ≥3.1**	40 (25.6)	30 (22.4)	10 (45.6)	
**Concomitant therapy, *n* (%)**				
** Oral 5-aminosalicylic acid**	131 (84.0)	110 (82.1)	18 (81.8)	>.95
** Topical 5-aminosalicylic acid**	22 (14.1)	16 (11.9)	6 (27.2)	.09
** Immunomodulator**	24 (15.4)	18 (13.4)	6 (27.3)	.11
** Biological agent**	42 (26.9)	34 (25.4)	8 (36.4)	.31
**Serum evaluation, median (IQR)**				
** White blood cell count, /μL**	5630 (4750-6770)	5680 (4785-6770)	5445 (4760-6455)	.74
** Hemoglobin, g/dL**	14.1 (12.7-15.3)	14.2 (12.7-15.4)	13.8 (12.6-14.6)	.62
** Albumin, g/dL**	4.5 (4.2-4.7)	4.5 (4.2-4.7)	4.3 (4.1-4.7)	.13
** C-reactive protein, mg/dL**	0.06 (0.04-0.16)	0.06 (0.04-0.16)	0.07(0.05-0.30)	.33

Abbreviation: IQR, interquartile range.

### 3.3. Primary analysis: still image-based relapse prediction

**Figure 4.**
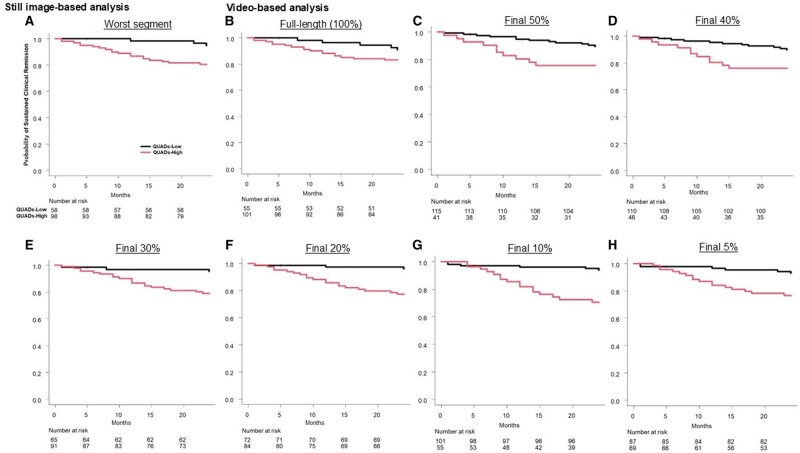
Kaplan–Meier estimates of the proportion of patients free from clinical relapse based on AI outputs. (A) Still image-based analysis (log-rank test: *P* = .01). Video-based analysis from: (B) full-length (100%) (log-rank test: *P* = .17), (C) final 50% segment (log-rank test: *P* = .02), (D) final 40% segment (log-rank test: *P* = .02), (E) final 30% segment (log-rank test: *P* < .01), (F) final 20% segment (log-rank test: *P* < .01), (G) final 10% segment (log-rank test: *P* < .01), and (H) final 5% segment (log-rank test: *P* < .01).

Still image-based QUAD scoring predicted clinical relapse within 24 months. Relapse occurred in 19.4% (19/98) of patients with QUAD ≥ 4 compared with 5.2% (3/58) of those with QUAD < 4 (*P* = .01). The AUC for relapse prediction was 0.67 (95% confidence interval [CI]: 0.57-0.76), with a sensitivity of 86.4% and specificity of 41.0%. Kaplan–Meier analysis demonstrated significant differences in relapse-free survival between groups (log-rank test, *P* = .01) (Figure 4a).

Histological assessment was available for all 156 patients in the prospective validation cohort. Histological activity, defined as a Geboes histological index ≥ 3.1, was significantly more frequent among patients with a worst still image-based QUAD score ≥ 4 than among those with a QUAD score < 4 (31.6% [31/98] vs 15.5% [9/58]; *P* = .04).

To evaluate the prognostic value of QUAD in relation to established relapse predictors, we performed a multivariable Cox proportional hazards analysis including MES, histological activity, maximum disease extent, use of biologics or Janus kinase (JAK) inhibitors, and the still image-based QUAD score. In this model, a worst MES ≥ 1 showed a trend toward association with clinical relapse (hazard ratio [HR], 3.22; 95% CI, 0.93-11.19; *P* = .07). Similarly, a worst still image-based QUAD score ≥ 4 showed a comparable trend toward association with relapse (HR, 3.16; 95% CI, 0.91-10.97; *P* = .07). Histological activity, maximum disease extent, and use of biologics or JAK inhibitors were not significantly associated with relapse in this model. (Table 2).

**Table 2.** Multivariable Cox proportional hazards analysis for clinical relapse within 24 months after colonoscopy.

Variable	Hazard ratio	95% CI	*P*-value
**MES ≥ 1**	3.22	0.93-11.20	.07
**Still image-based QUAD score ≥ 4**	3.16	0.91-10.97	.07
**Use of biologics or JAK inhibitors**	1.05	0.43-2.55	.92
**Geboes histological index ≥ 3.1**	2.29	0.84-6.23	.11
**Disease extent (extensive colitis)**	1.59	0.66-3.83	.30

Abbreviations: CI, confidence interval; JAK, Janus kinase; MES, Mayo Endoscopic Subscore; QUAD, Quantitative Ulcerative Colitis Assessment using Deep Learning.

### 3.4. Secondary exploratory analysis: video-based assessment of inflammatory extent

Still image-based and video-based analyses were evaluated separately because the former depended on endoscopist-selected images, whereas the latter used automated frame extraction from withdrawal videos. Automated full-length video analysis was performed to evaluate whether incorporating inflammatory extent improved relapse prediction. Full-length (100%) video analysis yielded an AUC of 0.62 (95% CI: 0.48-0.76), with a sensitivity of 63.6% and specificity of 61.2%. Segmental analyses revealed progressively improved predictive performance with increasing distal focus. Analyses restricted to the distal 50%, 40%, 30%, and 20% segments showed stepwise increases in discrimination (AUC range, 0.63-0.69) (Table 3). Predictive performance peaked at the distal 10% segment, which achieved the highest accuracy for relapse prediction (AUC, 0.73; 95% CI: 0.62-0.85; sensitivity, 72.7%; specificity, 70.9%). Further restriction to the distal 5% segment resulted in a slight decrease in performance (AUC, 0.71; 95% CI: 0.59-0.82), suggesting the presence of an optimal distal assessment window rather than a simple linear improvement with increasing distal restriction. Overall, incorporation of distal inflammatory extent improved relapse discrimination beyond both whole-colon video assessment and still image-based evaluation.

**Table 3.** Diagnostic performance for predicting clinical relapse within 24 months after colonoscopy.

	Area under the curve	95% confidence interval	QUADs cutoff value	Sensitivity (%)	Specificity (%)
**Still image-based analysis**	0.67	0.57-0.76	4	86.4	41.0
**Video-based analysis**					
** Full-length (100%)**	0.62	0.48-0.76	1.56	63.6	61.2
** Final 50%**	0.63	0.50-0.76	2.35	50.0	73.9
** Final 40%**	0.65	0.53-0.78	2.66	50.0	76.9
** Final 30%**	0.67	0.55-0.78	1.70	86.4	46.3
** Final 20%**	0.69	0.58-0.81	2.11	86.4	51.5
** Final 10%**	0.73	0.62-0.85	3.36	72.7	70.9
** Final 5%**	0.71	0.59-0.82	3.20	72.7	60.4

Abbreviation: QUADs, Quantitative Ulcerative Colitis Assessment using Deep Learning score.

## 4. Discussion

In this prospective study, we demonstrated that incorporating inflammatory extent into AI-based endoscopic assessment improves relapse risk stratification in UC. Compared with conventional severity-focused evaluation, analysis incorporating inflammatory extent provided higher predictive performance. Notably, distal inflammatory burden showed the strongest predictive performance in this cohort, suggesting that quantification of inflammatory extent may provide prognostic information beyond peak severity alone.

Our study showed that relapse prediction accuracy was higher when focusing on the distal colon rather than the entire colon. Specifically, analysis of the final 10% of the full-length colonoscopic video—corresponding to the distal sigmoid colon and rectum—outperformed conventional evaluations based on severity alone and whole-colon analysis. The AUC for this distal segment analysis was 0.73, compared with 0.68 for the single highest still-image score and 0.62 for whole-colon analysis. Although AI-based models have been recognized for enhancing objectivity in image interpretation,^15–18^ the subjectivity of image acquisition by endoscopists remains a critical challenge.^19^^,^^20^ Full-length video analysis may reduce, but does not eliminate, the frame-selection bias associated with endoscopist-selected still images because withdrawal technique, inspection time, mucosal cleaning, and image acquisition practices were not fully standardized.^21^ The cohort was not restricted to left-sided disease because 60.9% of patients had extensive colitis, suggesting that the superior predictive value of distal assessment was not driven solely by disease-distribution bias; however, the biological and technical explanations underlying this observation remain uncertain and should be interpreted cautiously. One possible explanation is that residual inflammation in the distal colon, particularly the distal sigmoid colon and rectum, may represent a sensitive marker of incomplete mucosal healing. By contrast, averaging inflammatory activity across the entire colon may dilute localized signals associated with subsequent relapse. Additionally, rectosigmoid segments are generally visualized more consistently during colonoscopy, which may improve the robustness of AI-based assessment. These hypotheses require further investigation and validation in independent cohorts.

This observation is biologically plausible because distal residual inflammation may represent a sensitive marker of persistent disease activity and has been linked to subsequent relapse in prior studies.^22–25^ These findings extend previous work evaluating cumulative inflammatory burden and AI-assisted endoscopic scoring systems in UC. Previous studies have focused primarily on lesion-level classification or treatment–response assessment,^12^^,^^13^ whereas our study addresses relapse prediction in patients already in clinical remission, representing a clinically relevant unmet need in treat-to-target management strategies. Within this evolving framework of AI-assisted endoscopic assessment, the aim of this study was not to replace conventional MES scoring but to explore whether incorporating inflammatory extent provides complementary prognostic information beyond severity-focused assessment. However, the predictive performance observed in this study was moderate (AUC, 0.67-0.73), suggesting that QUAD should be considered a complementary prognostic marker rather than a standalone clinical decision-making tool. Further integration with clinical, biochemical, and histologic markers, along with validation in independent cohorts, will be necessary before widespread clinical implementation.

Stidham et al.^12^ recently developed a video-based cumulative score that quantifies mucosal disease activity from the descending colon to the rectum, enabling a more efficient assessment of therapeutic response than the conventional MES. A major methodological distinction is that unlike the previous study, which focused on treatment response in patients experiencing clinical relapse, the still image-based component of the present study was designed to investigate clinical relapse exclusively in patients who were in clinical remission. Gutierrez-Becker et al.^13^ subsequently used full-colon datasets to create an AI scoring system that provides a spatially resolved evaluation of disease severity. To our knowledge, the present study is the first to investigate whether these novel indices are useful for predicting relapse during routine monitoring.

These findings highlight practical considerations for the clinical implementation of AI-based scoring. Reliable AI performance depends on high-quality colonoscopic imaging,^14^^,^^26^^,^^27^ and our system may further support image-quality standardization through real-time feedback mechanisms, although this requires further evaluation in future studies.

**Figure 5.**
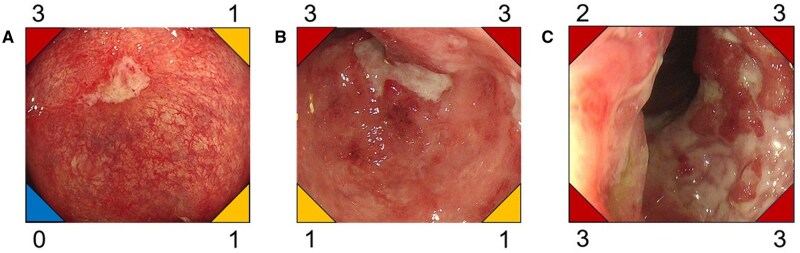
Representative images generated by the Quantitative Ulcerative Colitis Assessment using Deep Learning (QUAD) algorithm. (A) QUAD score 5, (B) QUAD score 8, and (C) QUAD score 11.

Complementing the concept of inflammatory extent across the colon, another distinctive feature of our model is its ability to capture patchy inflammatory patterns within a single endoscopic view, which may be particularly relevant in treated UC, where residual inflammation often becomes heterogeneous. Figure 5 illustrates images from the first component of this study that were interpreted by endoscopists as MES 3, with corresponding QUAD scores of 5, 8, and 11. However, when MES and QUAD were included in the same multivariable model, neither variable reached statistical significance, although both showed similar effect sizes. This finding suggests that QUAD and MES may capture partly overlapping aspects of residual endoscopic inflammation. In addition, the limited number of relapse events may have reduced the statistical power to distinguish the independent contribution of each endoscopic predictor. Therefore, the incremental value of QUAD beyond conventional endoscopic indices should be further evaluated in larger independent cohorts.

Several limitations should be acknowledged. Although multicenter data were used for model development, prospective validation was performed at a single specialized center, which may limit generalizability. The relatively small number of relapse events may limit the precision and stability of effect estimates and increase uncertainty around the observed predictive performance. Nevertheless, the use of standard white-light colonoscopy suggests potential applicability to routine clinical practice. Therapeutic strategies were not standardized and may have influenced relapse risk. Fecal calprotectin was not systematically available and could not be included in the multivariable model; therefore, the added value of QUAD in combination with biochemical markers requires further investigation. In addition, reliance on image quality may introduce bias despite automated filtering. Therefore, external validation in independent cohorts from different institutions and healthcare systems will be necessary to establish generalizability. Future prospective studies, including randomized comparisons of AI-assisted and standard endoscopic assessment strategies, are warranted to determine whether AI-guided risk stratification improves long-term clinical outcomes.

In conclusion, incorporating inflammatory extent into endoscopic assessment may enhance relapse risk stratification beyond conventional severity-focused evaluation. Our findings suggest that distal inflammatory burden represents a clinically relevant target for AI-assisted analysis during routine colonoscopy. However, given the moderate predictive performance observed, integration with clinical and non-endoscopic factors, along with validation in larger independent cohorts, will be essential before widespread clinical implementation.

## Supplementary Material

jjag115_Supplementary_Data

## Data Availability

The data supporting the findings of this study are not publicly available because of ethical and privacy restrictions, as participants did not consent to public data sharing.

## Patient and public involvement

Patients and/or the public were involved in the design, conduct, reporting, or dissemination plans of this research.
