# Supplementary material for: AI-based quantification of inflammatory extent for relapse prediction in ulcerative colitis: a prospective cohort study
Source: J Crohns Colitis. 2026 Jul 30;20(7):jjag115. doi: 10.1093/ecco-jcc/jjag115 (PMC13423237; doi:10.1093/ecco-jcc/jjag115)
Supplement: jjag115_Supplementary_Data [file jjag115_supplementary_data.zip › Supplementary Figure legend.docx]

**Supplementary Figure1: Schematic of the video segmentation workflow.**Withdrawal videos were sampled at 1 frame per second, and low-quality frames were excluded using the predefined quality-control algorithm. QUAD scores were assigned to each eligible frame. Mean QUAD scores were calculated for the full-length video and for the final 50%, 40%, 30%, 20%, 10%, and 5% portions of the video, which were defined according to withdrawal time. These segment-level scores were then used for relapse prediction analysis.
