# Supplementary figures and images for: AI-based quantification of inflammatory extent for relapse prediction in ulcerative colitis: a prospective cohort study
Source: J Crohns Colitis. 2026 Jul 30;20(7):jjag115. doi: 10.1093/ecco-jcc/jjag115 (PMC13423237; doi:10.1093/ecco-jcc/jjag115)

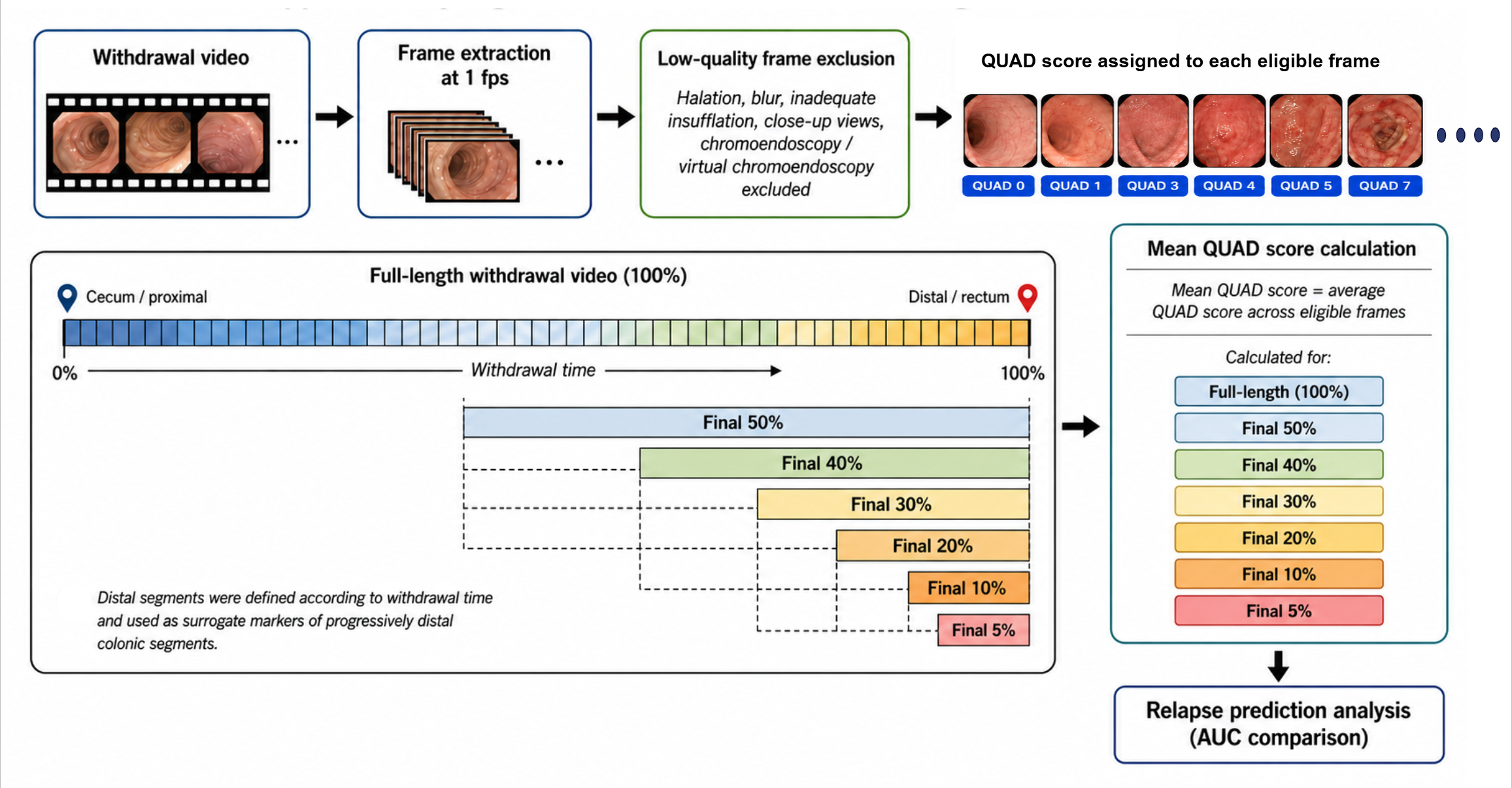

Supplement: jjag115_Supplementary_Data [file jjag115_supplementary_data.zip › Supplement Figure1.jpeg]
